# Study of laser actions by bird’s feathers with photonic crystals

**DOI:** 10.1038/s41598-021-81976-0

**Published:** 2021-01-28

**Authors:** Shih-Wen Chen, Jin-You Lu, Po-Han Tung, Ja-Hon Lin, Matteo Chiesa, Bing-Yi Hung, Thomas Chung-Kuang Yang

**Affiliations:** 1 Department of Chemical Engineering and Biotechnology, National Taipei University of Technology, 1, Sec. 3, Zhongxiao E. Rd., Taipei, 10608 Taiwan; 2 Laboratory for Energy and Nano Science, Department of Mechanical and Materials Engineering, Khalifa University, Abu Dhabi, United Arab Emirates; 3 Department of Electro-Optical Engineering, National Taipei University of Technology, 1, Sec. 3, Zhongxiao E. Rd., Taipei, 10608 Taiwan

**Keywords:** Lasers, LEDs and light sources, Laser material processing, Structural properties

## Abstract

Random lasers had been made by some biomaterials as light scattering materials, but natural photonic crystals have been rarely reported as scattering materials. Here we demonstrate the ability of natural photonic crystals to drive laser actions by sandwiched the feathers of the *Turquoise-Fronted Amazon* parrot and dye between two plastic films. Parrot feathers comprise abundant photonic crystals, and different color feathers compose of different ratios of the photonic crystal, which directly affect the feather reflectance. In this study, the multi-reflection scattering that occurred at the interface between the photonic crystal and gain media efficiently reduce the threshold; therefore, the more photonic crystal constitutes in the feathers; the lower threshold can be obtained. The random lasers can be easily made by the integration of bird feather photonic crystals and dye with a simple and sustainable manufacturing approach.

## Introduction

Random lasers (RLs) have attracted great attention during the past two decays because of their cost-effective and simple manufacturing process, free-angle emission, high radiance, and low spatial coherence, etc. The property of bright illuminations with low spatial coherence leads RLs to reach a significant achievement in imaging and display applications^1–6^. Except for lightning and imaging, RLs can also be used in optical sensors and optoelectronic devices^6–10^. 0D,2D, 3D heterostructures or polymers have been successfully developed for several optoelectronic applications^11–16^. Among them, 0D quantum dots (QDs), 2D graphdiyne, perovskite, metal–organic framework (MOF), and wrinkled polymers^10, 17–20^, have been investigated as the light scattering materials of RLs. To facilitate the practical applications of RLs, the strategy to reduce their threshold becomes a critical issue. Several methods were developed to reduce the threshold of RLs in combination with plasmonic nanoparticles, reflective mirrors, or fluorescence resonant energy transfer (FRET)^18–22^. For instance, Shen et al. demonstrated coherent Förster resonance energy transfer by mixed donor QDs and acceptor QDs to achieve the lasing threshold reduction^10^. Weng et al. studied that adding a patterned sapphire substrate efficiently reduced the lasing threshold from 2.55 to 0.15 μJ in CH_3_NH_3_PbBr_3_ perovskite thin films^18^. Hsiao et al. tuned the resonant energy transfer by different sizes of silver nanoparticles, and the threshold of random lasers from the dye-doped polymer is manipulated from 15.75 to 1.12 μJ^21^. Wan et al. reported plasmonic titanium nitride (TiN) nanoparticles enhanced low-threshold random lasing from dye-doped nematic liquid crystals^22^, the laser threshold decreases from 6.13 to 2.37 μJ/pulse when the number density of TiN nanoparticles increases from 5.613 × 10^10^/ml to 5.314 × 10^11^/ml. Furthermore, random lasers from bio-materials such as cicada wings, sands, or marine materials have been attracted by scientists for their natural textures and properties^23–26^. In this work, we will demonstrate that the neat arrangements of barbs and barbules in *Turquoise-Fronted Amazon* parrot feathers can form positive feedback loops, and natural photonic crystals in the barbs form multi-reflective mirrors can make laser actions with lower exciting energy. The random lasing from bird feathers with photonic crystals will be discussed by a facile method for the first time.

### Photonic crystals in parrot feathers

**Figure 1:**
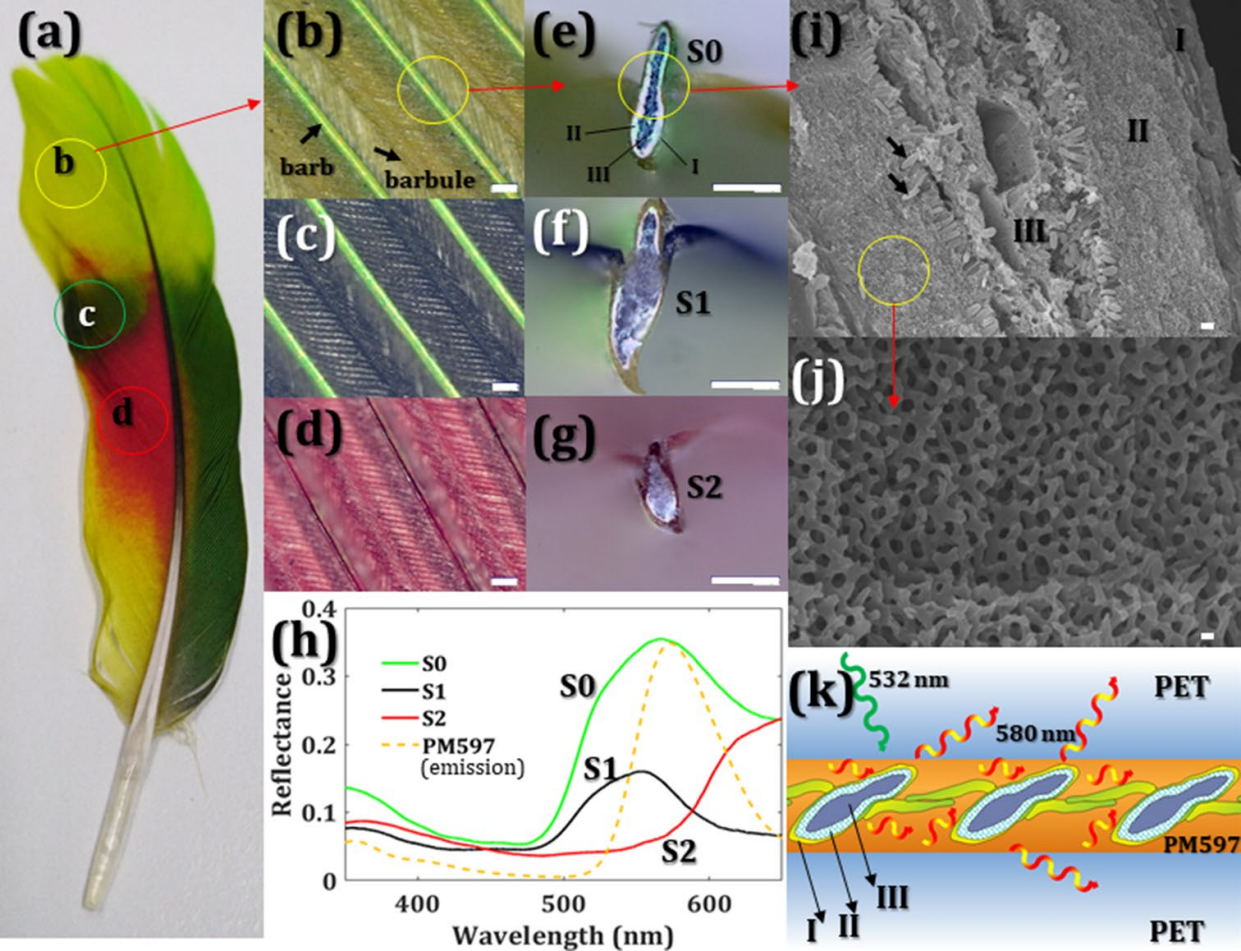
*Turquoise-Fronted Amazon* feather for random lasing. (**a**) The photo of a *Turquoise-Fronted Amazon* feather. (**b**)–(**d**) The images of yellow-green S0, dark-green S1, and red S2 feathers were zoomed in from circles b,c, and d in (**a**). (**e**)–(**g**) The cross-section views of (**b**)–(**d**). (**i**) The SEM image of (**e**), from I to III are the outer cortex, photonic crystals, and porous cells. The black arrow indicates the pigment. (**j**) The SEM image of photonic crystals. (**k**) Schematic illustration of the random laser sandwiched by two plastic layers. The scale bars are 100 μm in (**b**)–(**g**), 1 μm in (**i**), and 100 nm in (**j**).

In this study, parrot feathers with natural photonic crystals were selected as the scattering materials to act random lasing. Different from the iridescent coloration of butterfly wings, parrot feather barbs exhibit non-iridescent coloration^27, 28^ and the micro-geometry of parrot feathers is more complicated. Figure 1a shows a feather of *Turquoise-Fronted Amazon* parrot, which has yellow-green, dark-green, red, and yellow colors. Figures 1b–d reveal the barbs and their branches, barbules, in circles b, c, and d in Fig. 1a. From the cross-section views of barbs in Fig. 1e–g, the outer cortex, I, combines the keratin and pigments of pisttacofulvin, which manipulates the feather colors revealing red or yellow^28–31^. The middle layer, II, is photonic crystals that represent entirely white and the ratio of the photonic crystals (white area) shows yellow-green feather (S0) > dark-green feather (S1) > red feather (S2) in these images. The inner layer, III, combines a lot of holes and black pigments, melanin, forming porous spongy cells, which show black or gray colors. Besides, the barb size of the dark-green feather is the biggest and the barb size of the red feather is the smallest in these selected samples. The distance between barbules relates to the barb size, i.e. dark-green feather (S1) > yellow-green feather (S0) > red feather (S2). The scanning electron microscope (SEM) images reveal the structural details of the yellow-green outer cortex I, photonic crystals II, and the porous cells III with melanin (black arrow) in Fig. 1i. The outer cortex I, photonic crystals II, and porous cells III display yellow, white, and black (dark gray) colors, respectively, in Fig. 1e. Figure 1j is the SEM image of the photonic crystals. The coloration of the parrot feather is the result of the interaction of photonic crystals and pigments. The photonic crystal reflects most of the visible light, and different pigments absorb different wavelengths of visible light. Therefore, the feathers represent different colors^28, 31^. Figure 1h shows the reflectance of yellow-green feather S0, dark green feather S1, and red feather S2. The orange dotted line is the emission spectra of dye Pyrromethene (PM597, Exciton Inc.), which is opted as a gain media. It is worth noting that the area of the high reflectance of the feathers overlaps the main emission peak of PM597, i.e. 580 nm. Furthermore, from the reflectance spectra, the more photonic crystal contents, the higher reflectance the feather has. Figure 1k schematically illustrates the structure of the random lasing from parrot feather, where PM597 is coated on top of both sides of the barbs and its branches, barbule and embedded between two plastic films.

## Methods

**Figure 2:**
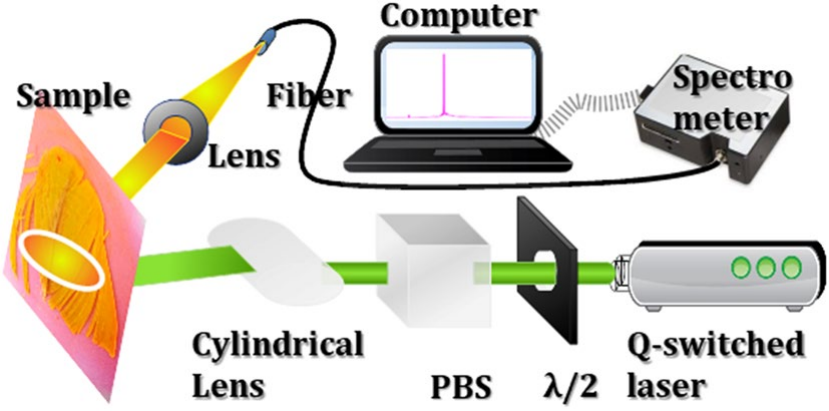
The illustration of the experimental setup and measurement of the random laser from parrot feathers.

The *Turquoise-Fronted Amazon* parrot feathers after molting were purchased from a pet shop in Taiwan. *Turquoise-Fronted Amazon* parrot is not the species in Washington Convention or a protected animal in Taiwan. To prepare the samples, the feathers were first cleaned with ethanol. The PM597 (0.01 g) was homogenous diluted in ethanol (5 mL) and then drop-coated on the different regimes of parrot feathers such as S0, S1, and S2. After drying, the dye covered feather were sealed within two polyethylene terephthalate (PET) films by a laminator. The setup of the random laser generation and measurement of the dye covered parrot feather is represented in Fig. 2. The pump source is a linearly polarized frequency-doubling Q-switched Nd: YAG laser (NL200 series, EKSPLA Inc.) with a central wavelength of 532 nm, a 10 Hz repetition rate, and 2.2 ns pulse duration. The exciting pump energy was controlled by the combination of a half-wave plate (λ/2) and a polarization beam splitter (PBS). A cylindrical lens with a 7 cm focal length provides sufficient area for exciting random lasing by extending the pump line stripe area. The emission signals from the samples were collected through fiber and measured by a spectrometer (HR-4000, Ocean Optics Inc.) with a 0.06 nm resolution. The angle between the pump beam and the lasing beam was around 15°–35°. The exciting area of the line stripe on the feather surface is around 0.0232 cm^2^, which is measured by the knife-edge method^32^.

## Results and discussion

**Figure 3:**
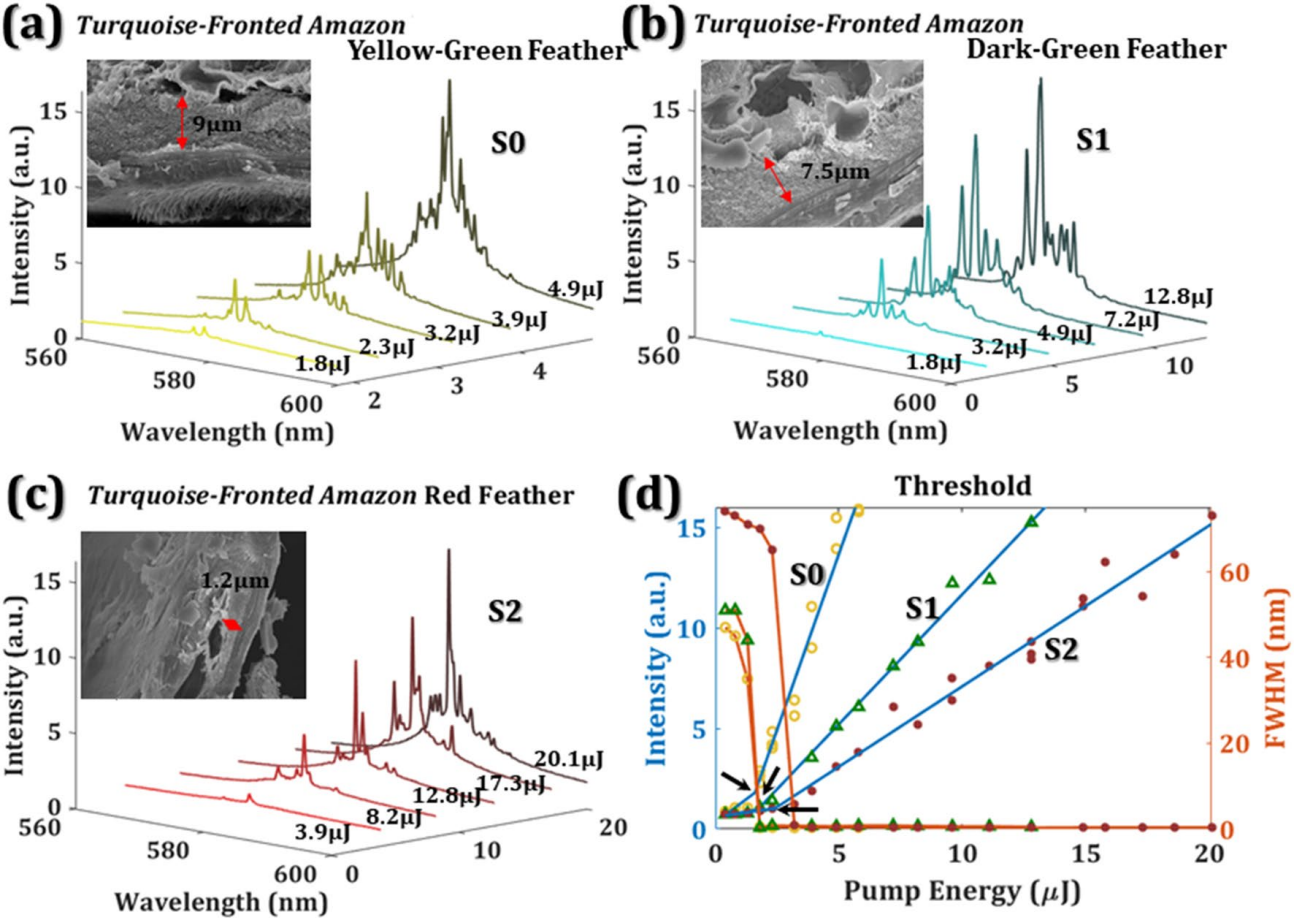
The emission spectra of *Turquoise-Fronted Amazon* (**a**) yellow-green feather S0, (**b**) dark-green feather S1, and (**c**) red feather S2. (**d**) The thresholds of the bird feather random lasers. Three black arrows indicate the threshold of 1.59 μJ, 1.7 μJ, and 2.4 μJ, respectively. The hollow circle, triangle, and solid circle represent the S0, S1, and S2, respectively.

The evolution of random lasing emission spectra from dye-covered *Turquoise-Fronted Amazon* parrot feather as a function of different pump energies are shown in Fig. 3. Different feather colors, such as yellow-green feather (S0), dark-green feather (S1), and red feather (S2), reveal different random lasing characteristics as shown in Fig. 3a–c. When the pump energies were below 1.8 μJ, only spontaneous emissions exist in Fig. 3a. When the excited energy increased, the quasi-periodic arrangement of textures and the branches of the parrot feathers efficiently scatter light to form many closed loops. Thus, random emission spikes, with aperiodic period and relatively large amplitude fluctuation, can be seen in Fig. 3a–c. The emission spikes from the dye covered feather located near the maximum emission peak of laser dye (PM597) around 580 nm. The output intensity as a function of pump intensity in Fig. 3d can be well fitted by two linear lines. The intersection of two fitting lines indicates the threshold of a laser device. The threshold energies of S0, S1, and S2 membranes are located at 1.59 μJ, 1.7 μJ, and 2.4 μJ, respectively. Comparing to S2, S0 and S1 have large photonic crystals as shown in the insets of Fig. 3a, b, where the thicknesses of the photonic crystal are 9 μm and 7.5 μm. The thickness of the photonic crystal of the red feather, S2, only has 1.2 μm. The threshold energies of S0 and S1 are lower than S2 due to the content of photonic crystals. The ratio of the photonic crystals also directly relates to the reflectance of the feathers in Fig. 1h, where the reflectance of S0 and S1 are both higher than S2 near the emission wavelength of PM597. From the results, we deduce that the yellow-green feather has the lowest threshold because of its highest reflectance near 580 nm, and the high reflectance is due to the highest content ratio of photonic crystals among these samples.

**Figure 4:**
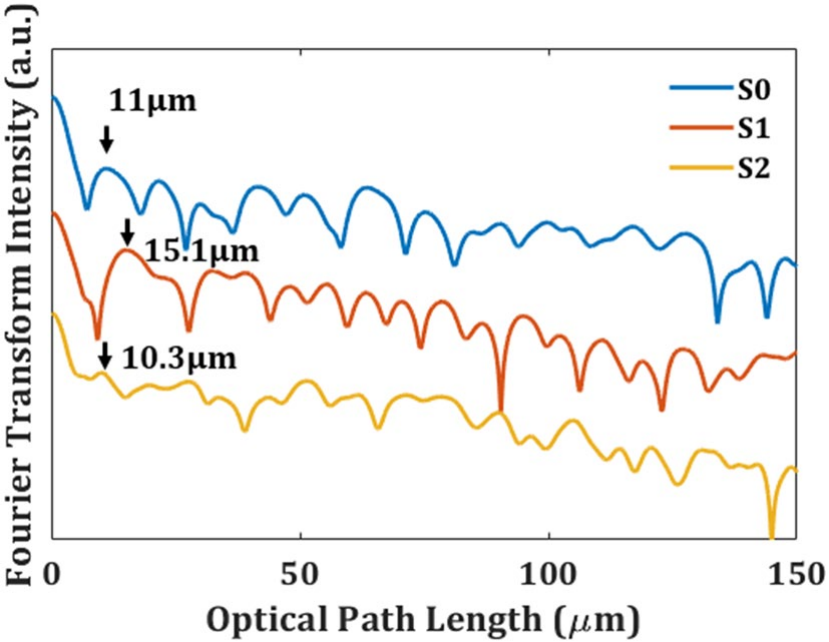
Fourier transform spectra versus optical path length of *Turquoise-Fronted Amazon* yellow-green feather S0, dark-green feather S1, and red feather S2.

No matter the pump light stripe is in parallel to the direction of barbs or it is perpendicular to the barbs, both of them can induce random lasing. To easily understand the optical length of the random lasers, Fourier transform spectra of the pump light strip in parallel to the barbs in Fig. 4 shows that S0, S1, and S2 have position peak of 11 μm, 15.1 μm, and 10.3 μm, respectively. The position peak d represents d = n L/π, where the refraction index is keratin alike, n = 1.53^33^. The reflective index of a dry PM597 film at 580 nm (n = 2.5) was fitted from an ellipsometry measurement. The optical lengths of S0, S1, and S2 are around 13.8 μm, 18.9 μm, and 12.9 μm, which are close to the distances between barbules.

**Figure 5:**
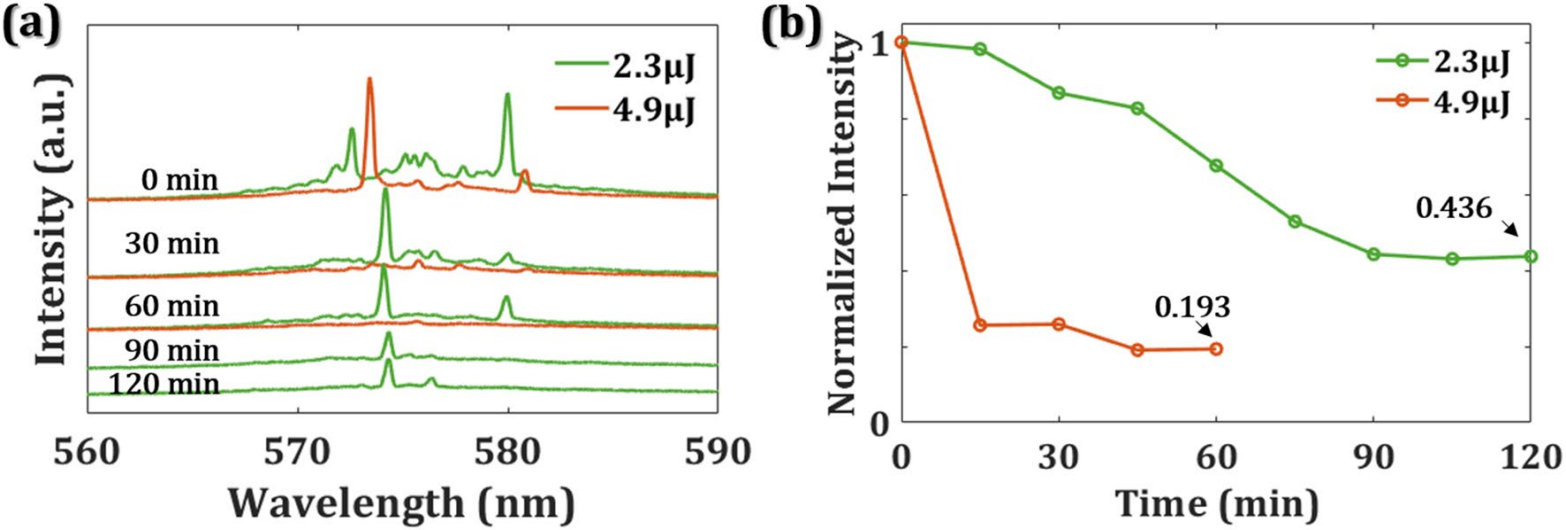
(**a**) Emission spectra of S0 with 2.3 μJ (green) and 4.9 μJ (red) pump energy for different exposure times. (**b**) the variation of normalized intensity from PL of S0 as a function of exposure time.

Furthermore, to understand the stability of the emission signals and the lifetime of sample S0, we record emission signals every fifteen minutes. The green and red lines in Fig. 5a, b indicate the emission intensity with the pump energy of 2.3 μJ and 4.9 μJ, respectively. There have no obvious spikes under the excitation of 4.9 μJ pump energy for one hour, but the spikes remain under the excitation of 2.3 μJ pump energy for 2 h. After two-hour excitation, the normalized intensity of RL reduces to 0.436 times of the initial intensity when the pump energy is 2.3 μJ. It has 0.193 times the initial intensity when the pump energy is 4.9 μJ and immediately drops a lot for 15 min. With proper pump energy, the bird laser can function well for more than 2 h. The micro/nano-structures on the feather will damage and break down the device if the exciting laser keeps irradiating on the surface with a high power pump laser. According to the results, we understand that the bird feather consists of keratin, one structural protein, which tolerates less exciting energy than chitin, the main material of butterfly wings^34^. However, the threshold of this framework is competitive with the metallic nanoparticles embedded in polymers^21^.

## Conclusions

In summary, we have adopted a facile method to build laser devices by incorporating natural photonic crystals within bird feathers as functional scattering media. The photonic crystal structure in feathers effectively generates coherent emissions owing to multi-scattering between feather and dye. From different photonic crystal contents in different color feathers, the random laser achieves a related low threshold of 1.59 μJ near the dye emission peak of 580 nm in the yellow-green feather, which has the highest content ratio of photonic crystals. Hence, the photonic crystals in the parrot function as multi-reflectors for laser action. The biology-based laser device not only has demonstrated the ability of the random laser action but also provided a sustainable material for future applications.

## Data Availability

The data that support the findings of this study are available from the corresponding author upon reasonable request.
